# Video-assisted thoracoscopic lobectomy versus open lobectomy in the treatment of large lung cancer: propensity-score matched analysis

**DOI:** 10.1186/s13019-021-01749-8

**Published:** 2022-01-08

**Authors:** Jeonghee Yun, Junghee Lee, Sumin Shin, Hong Kwan Kim, Yong Soo Choi, Jhingook Kim, Jae Il Zo, Young Mog Shim, Jong Ho Cho

**Affiliations:** Department of Thoracic and Cardiovascular Surgery, Samsung Medical Center, Sungkyunkwan University School of Medicine, 81 Irwon-ro, Gangnam-gu, Seoul, 06531 South Korea

**Keywords:** Large tumor, Non-small cell lung cancer, Video-assisted thoracoscopic surgery, Lobectomy

## Abstract

**Background:**

There are several concerns on thoracoscopic surgery for large tumors because of the increased risk of tumor cell spillage. This study aimed to compare perioperative outcomes and oncological validity between video-assisted thoracoscopic surgery (VATS) and open lobectomy for non-small cell lung cancer (NSCLC) with tumor size > 5 cm.

**Methods:**

We retrospectively reviewed 355 patients who underwent lobectomy with clinical N0 NSCLC with solid tumor component diameter > 5 cm between January 2009 and December 2016. Patients with tumor invading adjacent structures were excluded. The patients were divided into the VATS group (*n* = 132) and thoracotomy group (*n* = 223). Propensity score matching (1:1) was applied.

**Results:**

After propensity score matching, 204 patients were matched, and clinical characteristics of the two groups were well balanced. The VATS group was associated with a shorter length of hospital stay (6 days vs. 7 days; *P* < 0.001) than the thoracotomy group. There were no significant differences in the 5-year overall survival (71.5% in VATS vs. 64.4% in thoracotomy, *P* = 0.390) and 5-year recurrence-free survival (60.1% in VATS vs. 51.5% in thoracotomy, *P* = 0.210) between the two groups. The cumulative incidence of ipsilateral pleural recurrence was not significantly different between the two groups (12.0% in VATS vs. 7.9% in thoracotomy; *P* = 0.582).

**Conclusions:**

In clinical N0 NSCLC larger than 5 cm, VATS lobectomy resulted in shorter hospital stay and similar survival outcome compared to open lobectomy. Based on these results, VATS lobectomy is a valuable option in this subset of patients.

**Supplementary Information:**

The online version contains supplementary material available at 10.1186/s13019-021-01749-8.

## Background

Video-assisted thoracoscopic surgery (VATS) lobectomy is a commonly used approach in the treatment of early-stage non-small cell lung cancer (NSCLC). Numerous previous studies have shown that VATS lobectomy results in less pain, shorter hospital stay, and better postoperative quality of life compared with thoracotomy lobectomy, without compromising oncological outcomes [1–5]. The National Comprehensive Cancer Network guidelines recommend this approach in patients without anatomic or surgical contraindications [6].

With advances in surgical technique, the indication for VATS has been expanding to locally advanced NSCLC. However, surgeons still hesitate to perform thoracoscopic surgery on large tumors because thoracoscopic manipulation of large tumors is challenging and extraction of large tumors through a small incision may increase the risk of tumor rupture or cancer cell spillage [7]. Moreover, there is another concern regarding the adequacy of mediastinal lymph node assessment of VATS lobectomy, as larger tumors have more unexpected lymph node metastases [8–13].

Several studies have investigated the safety and efficacy of VATS lobectomy for NSCLC with tumor size > 5 cm. These studies showed that short- and long-term overall survival (OS) was not different between VATS and thoracotomy for patients with NSCLC with tumor size > 5 cm [14, 15]. However, these studies had a small sample size, heterogeneous population between treatment groups, and no information on cancer recurrence. Therefore, this study aimed to compare long-term OS and recurrence-free survival (RFS) between VATS lobectomy and thoracotomy lobectomy for NSCLC with tumor size > 5 cm after propensity score matching. Moreover, we measured the solid component diameter of the tumor to define clinical tumor size because this measurement better predicts prognosis than the total tumor size [16–20].

## Methods

### Study cohort

The data were obtained from a prospectively maintained institutional lung cancer database in Samsung Medical Center. A total of 5459 patients underwent curative lobectomy for primary NSCLC at Samsung Medical Center between January 2009 and December 2016. Of 4360 patients with clinical N0 NSCLC, patients with solid tumor component diameter > 5 cm on computed tomography (CT) were enrolled. Patients who had the following features were excluded from the study cohort: preoperative treatment with chemotherapy or radiation, primary tumor that invaded to adjacent structures (chest wall, pericardium, diaphragm, mediastinum, or esophagus), requirement of angioplasty or bronchoplasty, and another surgical approach other than thoracotomy or VATS (e.g., median sternotomy, transmanubrial approach). The study cohort was divided into the VATS group and thoracotomy group according to the initial intent (Additional file 1: Fig. 1). This retrospective study was approved by the ethics committee of the Samsung Medical Center, and informed consent was waived (Institutional Review Board number: SMC 2020-01-079).

### Staging workup

In this study, preoperative staging was performed in accordance with the American Joint Committee on Cancer (AJCC) Cancer Staging Manual, 7th edition, and patients’ TNM stage was reclassified according to the AJCC Cancer Staging Manual, 8th edition. The routine preoperative workup included chest CT, integrated whole-body 18F-fluorodeoxyglucose positron emission tomography and CT, and brain magnetic resonance imaging. In patients with suspicious nodal involvement, pathological confirmation of the suspected node was performed through endobronchial ultrasound-guided transbronchial needle aspiration or mediastinoscopy [21, 22].

### Surgical strategy and follow-up

Generally, VATS lobectomy was performed under single-lung anesthesia, using two ports and utility incision without rib spreading [1]. A 4-cm utility incision was made through the fifth or sixth intercostal space in the middle axillary line. Two additional ports were placed through the fourth or fifth intercostal space in the anterior and posterior axillary lines. However, details of the surgical techniques of VATS lobectomy varied among surgeons, as each surgeon has modified the technique according to their preferences.

The oncological principles remained the same regardless of the surgical approach: (1) the vessels and bronchi of the target lobe were individually dissected, (2) systematic lymph node dissection was regarded as mandatory, and (3) touching the lymph node itself and rupturing the capsule of the lymph node were avoided [1]. All specimens were placed into an impermeable bag and removed through the utility incision.

Patients were strictly followed every 3 months for the first 2 years postoperatively and every 6 months thereafter and underwent CT annually. Patients were also evaluated regularly via a history assessment, physical examination, blood tests, and chest radiography at each visit. Whole-brain CT or brain magnetic resonance imaging and other imaging techniques were performed as indicated, based on the patient’s symptoms.

### Statistical analysis

The primary outcome of this study was OS, defined as the time from surgery to death from any cause or last follow-up date. Secondary outcomes of this study were length of hospital stay, 30-day morbidity and mortality, ipsilateral pleural recurrence, and RFS, defined as time from surgery to recurrence or last recurrence surveillance confirming no evidence of recurrence. Locoregional recurrence was defined as that occurring within the surgical field, including the bronchial stump, pleural space, and regional lymph nodes. Distant recurrence was defined as that occurring beyond the surgical field that was not resected in the primary surgery.

Baseline characteristics and unadjusted outcomes were analyzed using the Wilcoxon rank sum test for continuous variables, and the chi-square test for categorical variables. Propensity score matching (1:1) was applied to reduce the effects of observed confounding between the two groups. *As surgical planning is decided based on the findings of the clinical staging assessment, we used clinical variables for propensity-score matched analysis.* Age, sex, tumor size, histology, pulmonary function test (forced expiratory volume in 1 s, diffusing capacity of lung for carbon monoxide), and Charlson comorbidity index were selected as covariates for propensity score analysis. Standardized mean differences were used to confirm a balanced matching result (Additional file 3: Table S1). When the standardized mean difference was < 0.1, we considered the matched values to be well balanced. For the matched cohort, baseline characteristics and perioperative outcomes were analyzed using the Wilcoxon signed rank test and McNemar test.

The Kaplan–Meier method and log-rank test were used to compare OS and RFS. Competing risk analysis and Gray’s method were used to assess and compare the cumulative incidence rate of recurrence in each recurrence pattern and ipsilateral pleural recurrence rate. Death or not-interested recurrence patterns were regarded as competing risks. A multivariate Cox proportional hazard analysis for OS was performed using covariates, which showed a P*-*value < 0.1 in univariate Cox proportional hazard analysis. A P*-*value < 0.05 was considered statistically significant.

## Results

### Study cohort

The study cohort consisted of 355 patients with clinical N0 NSCLC with solid tumor component diameters > 5 cm on chest CT. A total of 132 (37.2%) patients were assigned to the VATS group and 223 (62.8%) patients were assigned to the thoracotomy group. There were more women (27.3% in the VATS group vs 12.1% in the thoracotomy group; *P* < 0.001) and never-smokers (32.6% in the VATS group vs 12.1% in the thoracotomy group; *P* < 0.001) in the VATS group. The median clinical tumor size was 57 mm (interquartile range [IQR], 54–65) in the VATS group and 61 mm (IQR, 55–75) in thoracotomy group (*P* < 0.001). The most common histology was adenocarcinoma (66.7%) in the VATS group but squamous cell carcinoma (48.4%) in the thoracotomy group (*P* < 0.001).

After propensity score matching, 204 patients were available for analysis. The median clinical tumor size was 67 mm (IQR, 60–72) in the VATS group and 67 mm (IQR, 61–72) in the thoracotomy group (*P* = 0.871). The VATS and thoracotomy groups were well balanced for demographic and clinical characteristics (Table 1).

**Table 1:** Baseline characteristics

Variables	Unmatched cohort			Propensity score-matched cohort		
	Thoracotomy (*n* = 223)	VATS (*n* = 132)	*P *value	Thoracotomy (*n* = 102)	VATS (*n* = 102)	*P *value
Age	68 (61.0–73.0)	66 (59.8–71.0)	0.139	67 (61.0–72.0)	67 (60.0–72.0)	0.871
*Sex*						
Female	27 (12.1%)	36 (27.3%)	** < 0.001**	17 (16.7%)	18 (17.6%)	> 0.999
Male	196 (87.9%)	96 (72.7%)		85 (83.3%)	84 (82.4%)	
*Smoking*						
Never	27 (12.1%)	43 (32.6%)	** < 0.001**	19 (18.6%)	25 (24.5%)	0.829
Former	102 (45.7%)	55 (41.7%)		44 (43.1%)	47 (46.1%)	
Current	94 (42.1%)	34 (25.8%)		39 (38.2%)	30 (29.4%)	
*FEV1 (%)*						
≥ 80%	51 (22.9%)	20 (15.2%)	0.079	81 (79.4%)	84 (82.4%)	0.701
< 80%	172 (77.1%)	112 (84.9%)		21 (20.6%)	18 (17.6%)	
*DLCO (%)*						
≥ 80%	26 (11.7%)	8 (6.1%)	0.083	91 (89.2%)	94 (92.2%)	0.607
< 80%	197 (88.3%)	124 (93.9%)		11 (10.8%)	8 (7.8%)	
*CCI*						
0	146 (65.5%)	88 (66.7%)	0.627	66 (64.7%)	64 (62.7%)	0.809
1	49 (22.0%)	31 (23.5%)		26 (25.5%)	27 (26.5%)	
2	18 (8.1%)	6 (4.5%)		5 (4.9%)	5 (4.9%)	
≥ 3	10 (4.5%)	7 (5.3%)		5 (4.9%)	6 (5.9%)	
Tumor size	61 (55.0–75.0)	57(54.0–65.0)	** < 0.001**	59 (54.3–68.5)	59 (54.0–67.0)	0.777
*Location*						
RUL	53 (23.8%)	29 (22.0%)	0.060	20 (19.6%)	22 (21.6%)	0.106
RML	2 (0.9%)	7 (5.3%)		2 (2%)	7 (6.9%)	
RLL	54 (24.2%)	40 (30.3%)		25 (24.5%)	32 (31.4%)	
LUL	56 (25.1%)	26 (19.7%)		26 (25.5%)	20 (19.6%)	
LLL	58 (26.0%)	30 (22.7%)		29 (28.4%)	21 (20.6%)	
*Histology*						
ADC	90 (40.4%)	88 (66.7%)	** < 0.001**	62 (60.8%)	63 (61.8%)	> 0.999
SCC	108 (48.4%)	30 (22.7%)		28 (27.5%)	26 (25.5%)	
Others	25 (11.21%)	14 (10.61%)		12 (11.8%)	13 (12.7%)	

Data are presented as number (%), or median (interquartile range)

*FEV1* forced expiratory volume at 1 s, *DLCO* diffusing capacity for carbon monoxide, *CCI* Charlson comorbidity index, *ADC* adenocarcinoma, *SCC* squamous cell carcinoma, *VATS* video-assisted thoracic surgery

#### Perioperative outcomes

There were 10 conversions to thoracotomy from VATS: six were due to diffuse tight pleural adhesion, and four were due to anthracofibrotic lymph node around the pulmonary artery.

In the unmatched cohort, the median pathologic tumor size was 55 mm (IQR, 45–64.5) in the VATS group and 60 mm (IQR, 52.5–74) in thoracotomy group (*P* < 0.001). The VATS group had a shorter hospital stay than the thoracotomy group (6 days [5–8 days] vs 8 days [6–11 days]; *P* < 0.001). The 30-day morbidity and mortality did not show any significant differences between the two groups. In the propensity score-matched cohort, the median pathologic tumor size was 56 mm (IQR, 48–65) in the VATS group and 55.5 mm (IQR, 50–65) in the thoracotomy group (*P* = 0.348). The VATS group had a shorter hospital stay than the thoracotomy group (6 days [5–8 days] vs 7 days [6–10 days]; *P* < 0.001), whereas there were no differences in the 30-day morbidity and mortality (Table 2). The detailed information regarding complications within 30 days is shown in Table 3. In the unmatched cohort, the VATS group showed a lower incidence of pneumonia (0.8% vs 5.8%; *P* = 0.018) and arrhythmia (9.1% vs 17.9%; *P* = 0.023) than the thoracotomy group. Conversely, in the propensity score-matched cohort, the incidence of pneumonia was not significantly different (4.9% vs 0%; *P* = 0.064) between the two groups, but the incidence of arrhythmia was still significantly lower in the VATS group than in the thoracotomy group (8.1% vs 21.6%; *P* = 0.005).

**Table 2:** Perioperative outcomes

Variables	Unmatched cohort			Propensity score-matched cohort		
	Thoracotomy (*n* = 223)	VATS (*n* = 132)	*P *value	Thoracotomy (*n* = 102)	VATS (*n* = 102)	*P *value
Harvested LN number	22 (16.0–30.0)	16 (12.0–22.0)	** < 0.001**	22.5 (17.0–29.8)	16 (11.0–22.8)	** < 0.001**
*Bronchial margin*						
R0	222 (99.6%)	132 (100%)	> 0.999	111 (100%)	111 (100%)	> 0.999
R1	1 (0.4%)	0 (0.0%)		0 (0%)	0 (0%)	
Hospital length of stay	8 (6.0–11.0)	6 (5.0–8.0)	** < 0.001**	7 (6.0–10.0)	6 (5.0–8.0)	** < 0.001**
30-day morbidity	94 (42.2%)	42 (31.8%)	0.053	41 (40.2%)	32 (31.4%)	0.188
30-day mortality	2 (0.9%)	0 (0.0%)	0.147	1 (1%)	0 (0%)	> 0.999
Pathologic tumor size	60 (52.5–74)	55 (45–64.5)	< 0.001	55.5 (50–65)	56 (48–65)	0.348
*Pathologic N stage*						
pN0	169 (75.8%)	106 (80.3%)	0.147	72 (70.6%)	86 (84.3%)	0.143
pN1	29 (13.0%)	9 (6.8%)		18 (17.6%)	5 (4.9%)	
pN2	25 (11.2%)	16 (12.1%)		12 (11.8%)	11 (10.8%)	
Visceral pleural invasion	38 (17.0%)	28 (21.2%)	0.404	25 (24.5%)	23 (22.5%)	0.864
*Adjuvant treatment*						
No adjuvant treatment	93 (41.7%)	65 (49.2%)	0.204	43 (42.2%)	53 (52%)	0.262
Chemotherapy	110 (49.3%)	62 (47%)		52 (51%)	46 (45.1%)	
Radiotherapy	7 (3.1%)	1 (0.7%)		1 (1%)	1 (1%)	
Chemoradiotherapy	13 (5.8%)	4 (3%)		6 (5.9%)	2 (2%)	

Data are presented as number (%) or median (interquartile range)

**Table 3:** Complications within 30 days

Complications	Unmatched cohort			Propensity score-matched cohort		
	Thoracotomy (*n* = 223)	VATS (*n* = 132)	*P *value	Thoracotomy (*n* = 102)	VATS (*n* = 102)	*P *value
Number of patients	94 (42.2%)	42 (31.8%)	0.053	41 (40.2%)	32 (31.4%)	0.188
Number of events	119 (53.4%)	48 (36.4%)		92 (90.2%)	61 (59.8%)	
Pulmonary	41 (18.4%)	23 (17.4%)	0.820	16 (15.7%)	20 (19.6%)	0.585
Pneumonia/empyema	13 (5.8%)	1 (0.8%)	**0.018**	5 (4.9%)	0 (0%)	0.063
ARDS	7 (3.1%)	4 (3%)	> 0.999	2 (2%)	4 (3.9%)	0.687
Prolonged air leak/pneumothorax	16 (7.2%)	16 (12.1%)	0.116	8 (7.8%)	15 (14.7%)	0.167
Secretion retention/atelectasis	4 (1.8%)	0 (0%)	0.301	1 (1%)	0 (0%)	> 0.999
Others*	1 (0.4%)	2 (1.5%)	0.558	0 (0%)	1 (1%)	> 0.999
Cardiac	41 (18.4%)	12 (9.1%)	**0.018**	21 (20.6%)	6 (5.9%)	**0.003**
Arrhythmia	40 (17.9%)	12 (9.1%)	**0.023**	20 (19.6%)	6 (5.9%)	**0.004**
MI	1 (0.4%)	0 (0%)	> 0.999	1 (1%)	0 (0%)	> 0.999
CVA	6 (2.7%)	1 (0.8%)	0.265	3 (2.9%)	1 (1%)	0.625
Bleeding	1 (0.4%)	2 (1.5%)	0.558	0 (0%)	1 (1%)	> 0.999
Wound dehiscence	4 (1.8%)	1 (0.8%)	0.655	3 (2.9%)	1 (1%)	0.625
Others**	26 (11.7%)	9 (6.8%)	0.139	12 (11.8%)	6 (5.9%)	0.180

Data are presented as number (%)

*ARDS* acute respiratory distress syndrome, *MI* myocardial infarction

*Other pulmonary complications include pleural effusion and acute exacerbation of chronic obstructive pulmonary disease

**Other complications include delirium, chylothorax, ileus, vocal cord palsy, and urinary tract infection

### Survival outcomes

**Fig. 1:**
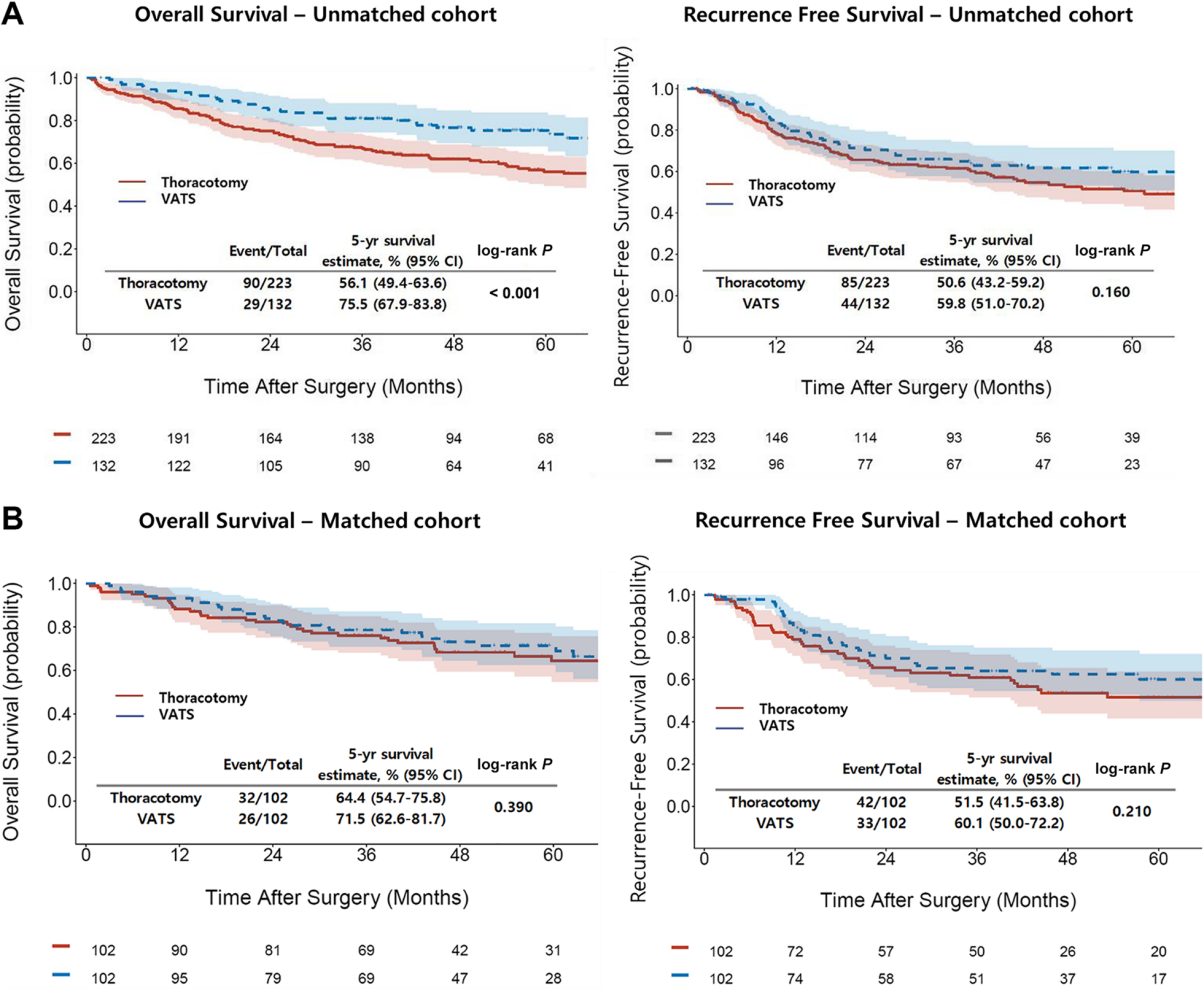
Survival outcomes. Kaplan–Meier estimation of VATS group showed better overall survival and similar recurrence-free survival than the thoracotomy group in unmatched cohort. **a** After propensity score matching (1:1), the survival curve showed that there were no significant differences in overall and recurrence-free survivals between the VATS and thoracotomy groups. **b** VATS, video-assisted thoracoscopic surgery; CI, confidence interval

The median follow-up period of the entire cohort was 80 months. At the completion of the study, 134 patients died, and 130 patients had recurrence during the follow-up. In the VATS group, 33 patients died, and 44 patients had recurrence, while, in the thoracotomy group, 101 patients died, and 86 patients had recurrence. The 5-year OS rates were 75.5% in the VATS group and 56.1% in the thoracotomy group. The log-rank test showed that the VATS group had significantly superior OS compared with the thoracotomy group (*P* < 0.001). In contrast, the 5-year RFS rates was not significantly different between the groups (59.8% in the VATS group vs. 50.6% in the thoracotomy group, *P* = 0.160) (Fig. 1a).

After propensity score matching, the 5-year OS rates were 71.5% in the VATS group and 64.4% in the thoracotomy group. The 5-year RFS rates were 60.1% in the VATS group and 51.5% in the thoracotomy group. There were no significant differences in the OS and RFS between the VATS and thoracotomy groups (OS, *P* = 0.390; RFS, *P* = 0.210) (Fig. 1b). Additional survival analysis was performed in the subgroups of the matched cohort. In the subgroups stratified by pathologic lymph node metastasis, there were no statistically significant differences in OS between the VATS and thoracotomy groups (Additional file 2: Fig. 2a). Similarly, no significant survival differences were observed in subgroups stratified by tumor histology (Additional file 2: Fig. 2b).

In the multivariate Cox proportional hazard model analysis for OS, surgical approach (VATS vs. thoracotomy) was not an independent risk factor for survival (hazard ratio, 0.76; 95% confidence interval, 0.51–1.15; *P* = 0.195) after adjusting for sex, age, Charlson comorbidity index, pathologic tumor size, pathologic lymph node metastasis, tumor histology, and adjuvant therapy (Table 4).

**Table 4:** Multivariable Cox proportional hazard model for OS in unmatched cohort

Variable	Univariable analysis				Multivariable analysis				
	Hazard ratio	95% CI		*P* value	Hazard ratio		95% CI		*P *value
VATS (vs. thoracotomy)	0.56	0.38	0.82	**0.003**	0.81		0.54	1.23	0.331
Sex, male (vs. female)	2.23	1.28	3.88	**0.005**	1.65		0.93	2.95	0.087
Age (per year)	1.05	1.03	1.08	** < 0.001**	1.04		1.02	1.07	** < 0.001**
CCI ≥ 2 (vs. CCI < 2)	2.10	1.34	3.30	**0.001**	1.72		1.09	2.74	**0.021**
FEV1 ≥ 80% (vs. < 80%)	1.02	0.66	1.56	0.935					
DLCO ≥ 80% (vs. < 80%)	0.77	0.45	1.32	0.349					
Pathologic tumor size	1.02	1.01	1.03	** < 0.001**	1.02		1.01	1.03	** < 0.001**
Pathologic N + (vs. pN0)	1.90	1.32	2.75	**0.001**	2.33		1.60	3.40	** < 0.001**
Histology (ref = ADC)				**0.009**					
SCC	1.65	1.16	2.36		1.13		0.77	1.65	0.542
Others	0.87	0.46	1.66		0.86		0.44	1.65	0.643
Adjuvant treatment	0.65	0.47	0.92	**0.013**	0.69		0.47	1.03	0.071

Statistically significant variates (*P* < 0.1) in the univariable analysis were used as covariates for multivariable analysis

*VATS* video-assisted thoracic surgery, *CCI* Charlson comorbidity index, *FEV1* forced expiratory volume at 1 s, *DLCO* diffusing capacity for carbon monoxide, *ADC* adenocarcinoma, *SCC* squamous cell carcinoma

### Recurrence patterns

**Fig. 2:**
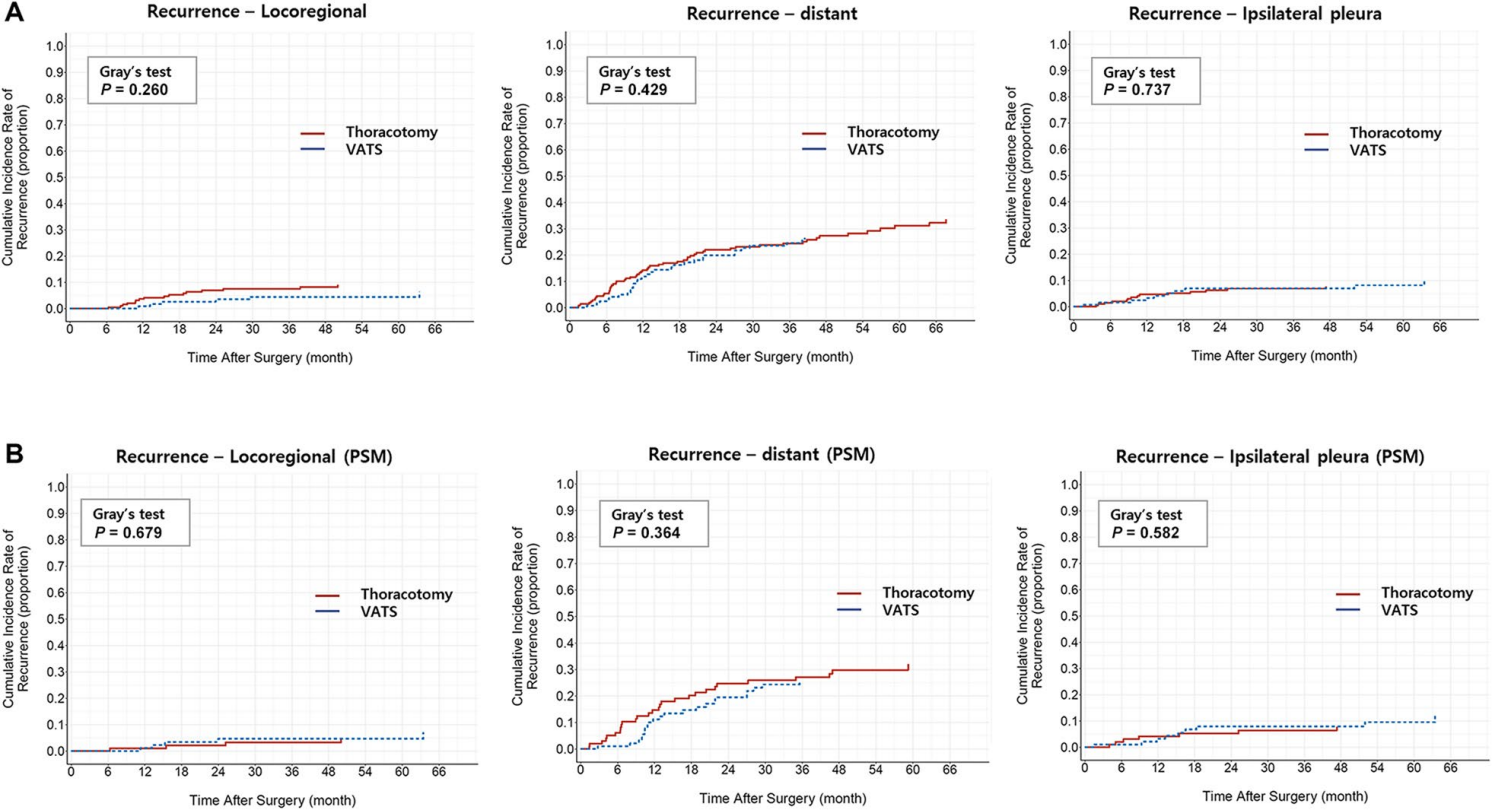
Cumulative incidence of recurrence. Cumulative incidence of locoregional, distant, and ipsilateral pleural recurrences were not different between VATS and thoracotomy groups in both unmatched (**a**) and matched cohorts (**b**)

Of the 130 patients with recurrence, locoregional recurrence occurred in 22 patients, distant recurrence occurred in 86 patients, and ipsilateral pleural recurrence occurred in 24 patients (Additional file 3: Table 2). The cumulative incidence rate (CIR) of locoregional recurrence at 5 years was 6.4% in the VATS group and 9.1% in the thoracotomy group (Gray’s test, *P* = 0.260). The CIR of distant metastasis was similar between the two groups (26.6% vs 32.3%; *P* = 0.429). No differences were observed in the CIR of locoregional recurrence and distant metastasis after propensity matching (*P* = 0.679 and *P* = 0.364, respectively) (Fig. 2). The VATS group did not show an increased risk of ipsilateral pleural recurrence in the unmatched cohort (10.1% in the VATS group vs. 7.7% in the thoracotomy group, *P* = 0.737) and matched cohort (12.0% in the VATS group vs. 7.9% in the thoracotomy group, 0.582).

## Discussion

Thoracoscopic surgery for large tumor is challenging because a large tumor interferes with the surgeon’s delicate movements, which can sometimes lead to vessel injury or tumor rupture. It is technically more difficult to obtain sufficient resection margin in VATS than in thoracotomy when a large tumor is centrally located. Moreover, extraction of the large tumor via small incision can crush the tumor or tear the extraction bag. Theoretically, these may increase ipsilateral pleural recurrence or locoregional recurrence. However, the current study showed that VATS lobectomy did not increase ipsilateral pleural recurrence or locoregional recurrence compared to thoracotomy, and the 5-year RFS was not different between the two groups. Moreover, VATS lobectomy demonstrated benefit in the early postoperative period. The length of hospital stay was significantly reduced, and postoperative pneumonia and arrythmia less occurred in the VATS group. It shows that the technical difficulty of VATS lobectomy did not adversely affect the postoperative and long-term survival outcomes in large lung tumor.

Another concern on VATS lobectomy for large lung tumor is the adequacy of mediastinal lymph node assessment. Although many previous studies have shown that the number of removed lymph nodes by VATS was not inferior to open thoracotomy in early lung cancer [4, 23–27] and a recent study showed no difference in pathological nodal upstaging rate between VATS and thoracotomy even in clinical N1 NSCLC [28], concerns still remain on the adequacy of thoracoscopic lymph node assessment in clinical stage II lung cancer. In this study, although more lymph nodes were harvested in the thoracotomy group, the number of harvested lymph nodes in the VATS group was sufficient to meet the requirement of the 8th edition of the AJCC Cancer Staging Manual, which recommends removal of at least six lymph nodes [29]. Moreover, the pathologic nodal upstaging rate and incidence of regional lymph node recurrence were not statistically different between the VATS and thoracotomy groups. Moreover, long-term survival was not different between VATS and thoracotomy groups in the subgroup stratified by pathologic nodal upstaging (Additional file 2: Fig. 2a). It indicates that thoracoscopic lymph node dissection does not reduce the quality of lymph node dissection in large lung cancer.

The notable point in this study is that we measured clinical tumor size according to the TNM cancer staging system 8th edition that was revised to measure the solid tumor diameter rather than the total diameter to describe the T stage. Previous studies measured total diameter to determine the tumor size and had no information on consolidation to tumor ratio. However, it can create an error because solid tumor has a worse prognosis than part-solid or pure ground glass opacity tumor even if they have the same total diameter, and the treatment group including more solid tumor may have worse survival outcome. By measuring the solid diameter for tumor size and using propensity score analysis, the compounding factors were better controlled compared to those in previous studies. Moreover, the current study has the largest number of patients (*n* = 355) among related studies.

The present study has several limitations. First, the retrospective nature of this study allows the possibility of uncontrolled confounding, although propensity score matching was used to balance the variables that may influence the outcomes between the groups. Second, selection of surgical approach depends on the extent of disease in general, but the individual surgeon’s preferences may also have influenced the decision. Lastly, the results of this study are results from experienced surgeon and cannot be generalized to beginners. Despite these limitations, this study showed the efficacy of VATS lobectomy in clinical stage II lung cancer and may be used as a reference for larger prospective studies.

## Conclusions

In patients with clinical N0 NSCLC with tumor diameter > 5 cm, VATS lobectomy demonstrated a shorter hospital stay and similar survival outcome compared with open lobectomy after propensity score matching. Based on these results, VATS lobectomy is a valuable option in this subset of patients.

## Supplementary Information

**Additional file 1: Figure 1**. Flow diagram of study cohort.

**Additional file 2: Figure 2**. Overall survival of the subgroup in the propensity matched cohort. (a) Overall survival of pN0 patients in the matched cohort. (b) Overall survival of pN+ patients in the matched cohort. (c) Overall survival of patients with adenocarcinoma in the matched cohort. (d) Overall survival of patients with squamous cell carcinoma in the matched cohort.

**Additional file 3: Table 1**. Standard mean difference of matched variables. **Table 2** Pattern of recurrence.

## Abbreviations

VATS: Video-assisted thoracoscopic surgery
NSCLC: Non-small cell lung cancer
RFS: Recurrence-free survival
OS: Overall survival
AJCC: American Joint Committee on Cancer
FEV1: Forced expiratory volume in 1 s
DLCO: Diffusing capacity of lung for carbon monoxide
CIR: Cumulative incidence rate
CCI: Charlson comorbidity index
ADC: Adenocarcinoma
SCC: Squamous cell carcinoma
ARDS: Acute respiratory distress syndrome
MI: Myocardial infarction
