# Supplementary figures and images for: Video-assisted thoracoscopic lobectomy versus open lobectomy in the treatment of large lung cancer: propensity-score matched analysis
Source: J Cardiothorac Surg. 2022 Jan 8;17:2. doi: 10.1186/s13019-021-01749-8 (PMC8742315; doi:10.1186/s13019-021-01749-8)

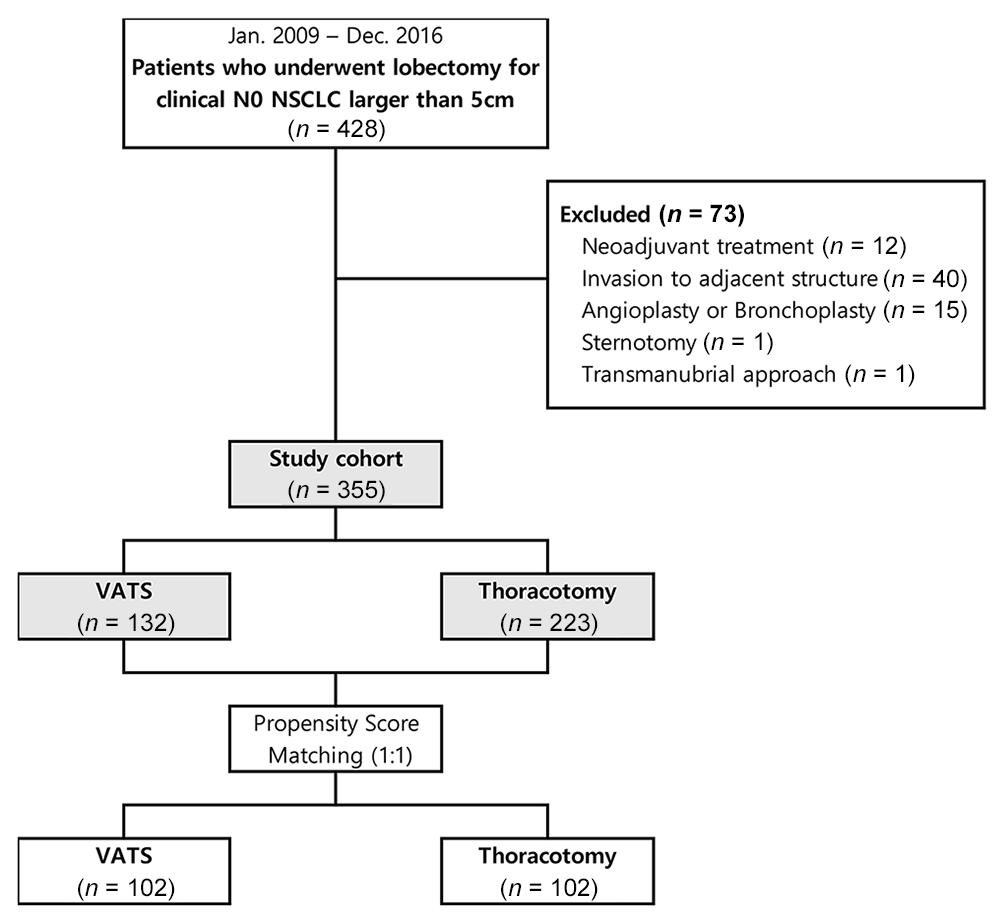

Supplement: Supplementary file 1 — Additional file 1: Figure 1. Flow diagram of study cohort. [file 13019_2021_1749_MOESM1_ESM.tif]

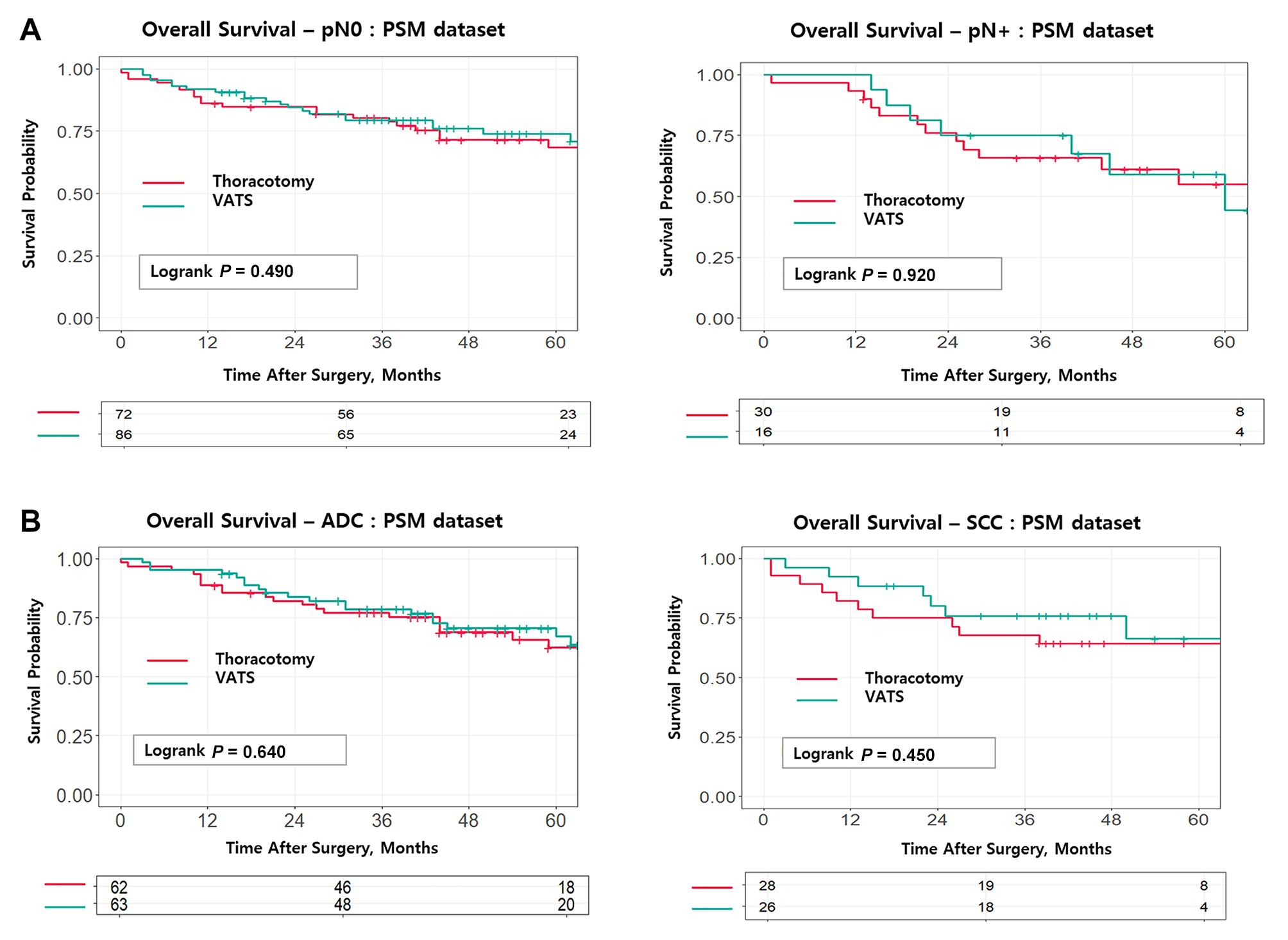

Supplement: Supplementary file 2 — Additional file 2: Figure 2. Overall survival of the subgroup in the propensity matched cohort. (a) Overall survival of pN0 patients in the matched cohort. (b) Overall survival of pN+ patients in the matched cohort. (c) Overall survival of patients with adenocarcinoma in the matched cohort. (d) Overall survival of patients with squamous cell carcinoma in the matched cohort. [file 13019_2021_1749_MOESM2_ESM.tif]
