# Supplementary material for: Video-assisted thoracoscopic lobectomy versus open lobectomy in the treatment of large lung cancer: propensity-score matched analysis
Source: J Cardiothorac Surg. 2022 Jan 8;17:2. doi: 10.1186/s13019-021-01749-8 (PMC8742315; doi:10.1186/s13019-021-01749-8)
Supplement: Supplementary file 3 — Additional file 3: Table 1. Standard mean difference of matched variables. Table 2 Pattern of recurrence. [file 13019_2021_1749_MOESM3_ESM.docx]

| **Supplementary Table 1** Standard mean difference of matched variables | | | | | | | | |
| --- | --- | --- | --- | --- | --- | --- | --- | --- |
| Variables | Unmatched cohort | | | | Propensity score-matched analysis | | | |
|  | Thoracotomy  (n=223) | VATS  (n=132) | SMD | *P*-value | Thoracotomy  (n=102) | VATS  (n=102) | SMD | *P*-value |
| Age | 68 (61.0–73.0) | 66 (59.8–71.0) | 0.131 | 0.139 | 67 (61.0–72.0) | 67 (60.0–72.0) | 0.02 | 0.871 |
| Sex |  |  | 0.388 | **<0.001** |  |  | 0.03 | >0.999 |
| Female | 27 (12.1%) | 36 (27.3%) |  |  | 17 (16.7%) | 18 (17.6%) |  |  |
| Male | 196 (87.9%) | 96 (72.7%) |  |  | 85 (83.3%) | 84 (82.4%) |  |  |
| FEV1 (%) |  |  |  |  |  |  | 0.07 | 0.701 |
| ≥80% | 51 (22.9%) | 20 (15.2%) |  | 0.079 | 81 (79.4%) | 84 (82.4%) |  |  |
| <80% | 172 (77.1%) | 112 (84.9%) |  |  | 21 (20.6%) | 18 (17.6%) |  |  |
| DLCO (%) |  |  | 0.297 | 0.083 |  |  | 0.03 | 0.607 |
| ≥80% | 26 (11.7%) | 8 (6.1%) |  |  | 91 (89.2%) | 94 (92.2%) |  |  |
| <80% | 197 (88.3%) | 124 (93.9%) |  |  | 11 (10.8%) | 8 (7.8%) |  |  |
| CCI |  |  | 0.15 | 0.627 |  |  | 0.05 | 0.809 |
| 0 | 146 (65.5%) | 88 (66.7%) |  |  | 66 (64.7%) | 64 (62.7%) |  |  |
| 1 | 49 (22.0%) | 31 (23.5%) |  |  | 26 (25.5%) | 27 (26.5%) |  |  |
| 2 | 18 (8.1%) | 6 (4.5%) |  |  | 5 (4.9%) | 5 (4.9%) |  |  |
| ≥3 | 10 (4.5%) | 7 (5.3%) |  |  | 5 (4.9%) | 6 (5.9%) |  |  |
| Tumor size | 61 (55.0–75.0) | 57(54.0–65.0) | 0.37 | **<0.001** | 59 (54.3–68.5) | 59 (54.0–67.0) | 0.03 | 0.777 |
| Histology |  |  | 0.585 | **<0.001** |  |  | 0.05 | >0.999 |
| ADC | 90 (40.4%) | 88 (66.7%) |  |  | 62 (60.8%) | 63 (61.8%) |  |  |
| SCC | 108 (48.4%) | 30 (22.7%) |  |  | 28 (27.5%) | 26 (25.5%) |  |  |
| Others | 25 (11.21%) | 14 (10.61%) |  |  | 12 (11.8%) | 13 (12.7%) |  |  |

FEV1, forced expiratory volume at 1 s; DLCO, diffusing capacity for carbon monoxide; CCI, Charlson comorbidity index; ADC, adenocarcinoma; SCC, squamous cell carcinoma.

| **Supplementary Table 2** Pattern of recurrence | | | | | |
| --- | --- | --- | --- | --- | --- |
| Variables | Unmatched cohort | |  | Propensity score-matched cohort | |
|  | Thoracotomy  (n=223) | VATS  (n=132) |  | Thoracotomy  (n=102) | VATS  (n=102) |
| Recurrent event during follow-up |  |  |  |  |  |
| Locoregional | 16 (7.2%) | 6 (4.5%) |  | 4 (3.9%) | 5 (4.9%) |
| Distant | 56 (25.1%) | 30 (22.7%) |  | 28 (27.5%) | 22 (21.6%) |
| Combined | 14 (6.3%) | 8 (6.1%) |  | 10 (9.8%) | 6 (5.9%) |
| Ipsilateral pleura | 14 (6.3%) | 10 (7.6%) |  | 7 (6.9%) | 9 (8.8%) |
|  |  |  |  |  |  |
| CIR at 5 years |  |  |  |  |  |
| Locoregional | 9.1% | 6.4% |  | 4.9% | 7.1% |
| Distant | 32.3% | 26.6% |  | 32.0% | 25.6% |
| Ipsilateral pleura | 7.7% | 10.1% |  | 7.9% | 12.0% |

**
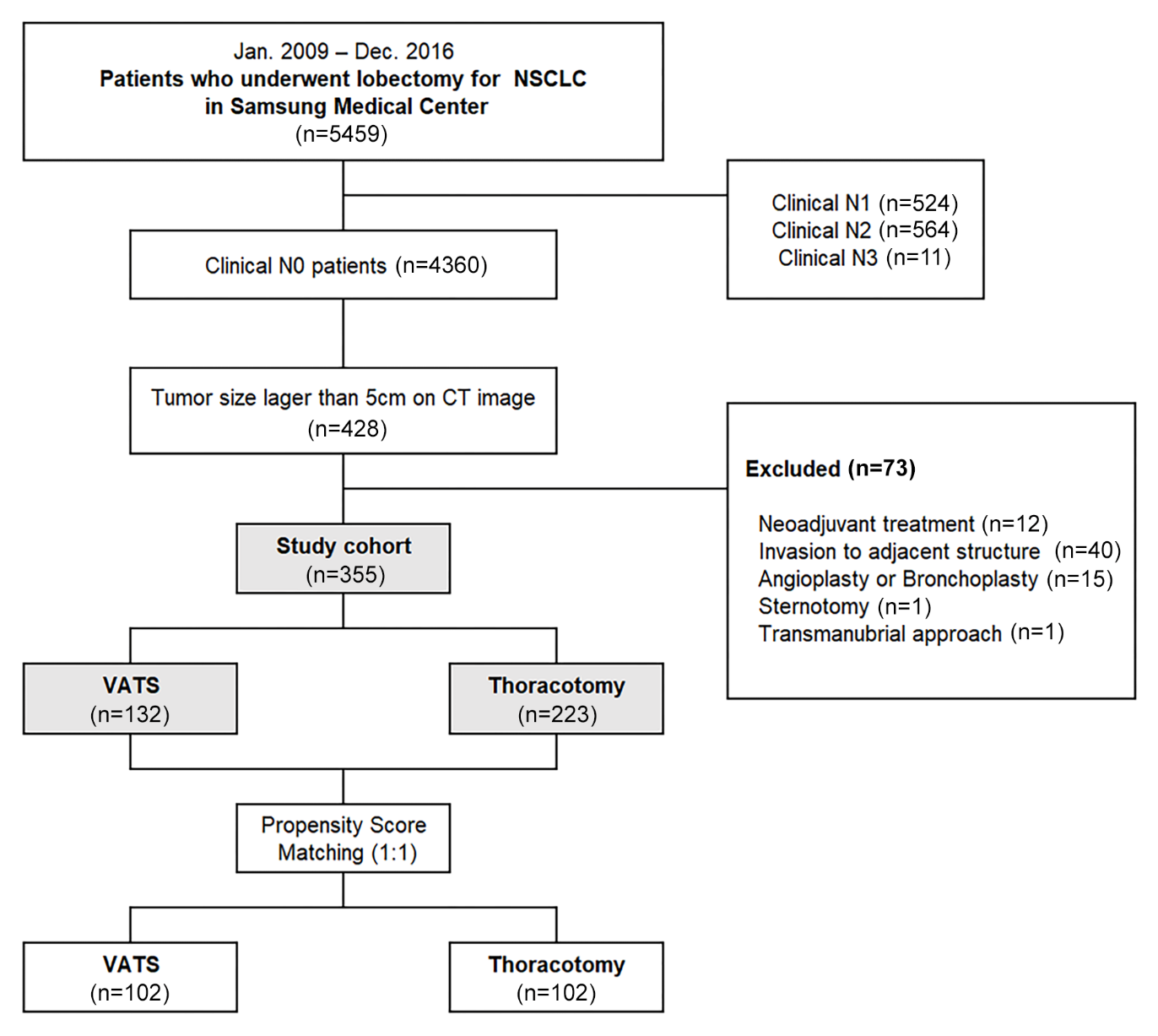
**

**Supplementary Figure 1** Flow diagram of study cohort.

**
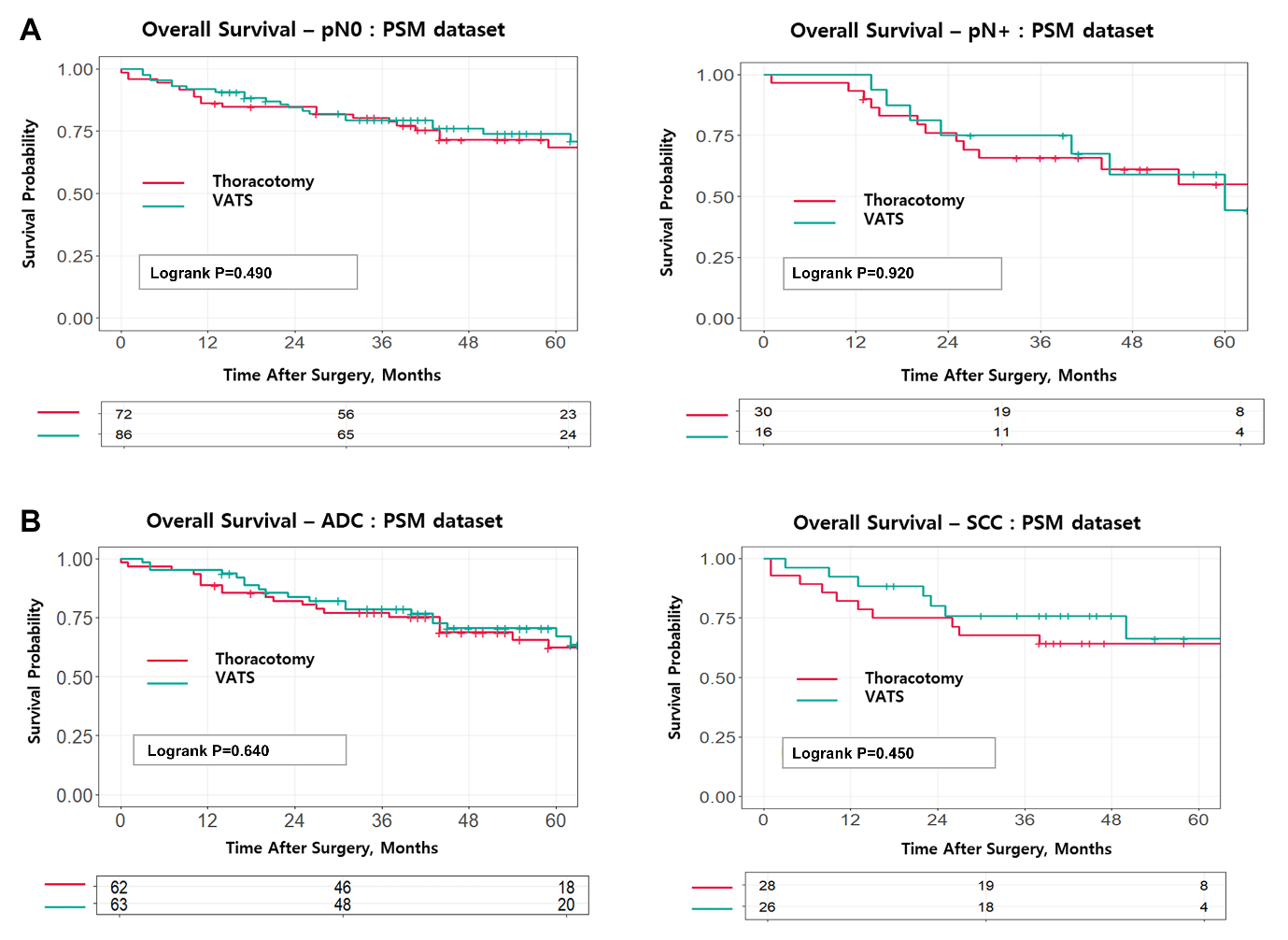
**

**Supplementary Figure 2** Overall survival of the subgroup in the propensity matched cohort. (A) Overall survival of pN0 patients in the matched cohort. (B) Overall survival of pN+ patients in the matched cohort. (C) Overall survival of patients with adenocarcinoma in the matched cohort. (D) Overall survival of patients with squamous cell carcinoma in the matched cohort.
